# Segmentectomy versus lobectomy in younger patients with early-stage non-small cell lung cancer

**DOI:** 10.1093/icvts/ivaf024

**Published:** 2025-02-10

**Authors:** Atsushi Kamigaichi, Takahiro Mimae, Norifumi Tsubokawa, Yoshihiro Miyata, Yujin Kudo, Takuya Nagashima, Hiroyuki Ito, Norihiko Ikeda, Morihito Okada

**Affiliations:** Department of Surgical Oncology, Hiroshima University, Hiroshima, Japan; Department of Surgical Oncology, Hiroshima University, Hiroshima, Japan; Department of Surgical Oncology, Hiroshima University, Hiroshima, Japan; Department of Surgical Oncology, Hiroshima University, Hiroshima, Japan; Department of Surgery, Tokyo Medical University, Tokyo, Japan; Department of Thoracic Surgery, Kanagawa Cancer Center, Yokohama, Japan; Department of Thoracic Surgery, Kanagawa Cancer Center, Yokohama, Japan; Department of Surgery, Tokyo Medical University, Tokyo, Japan; Department of Surgical Oncology, Hiroshima University, Hiroshima, Japan

**Keywords:** young, non-small cell lung cancer, lobectomy, segmentectomy

## Abstract

**OBJECTIVES:**

Despite clinical trials supporting the efficacy of segmentectomy for early-stage non-small cell lung cancer (NSCLC), a previous report indicated its limited efficacy in younger patients, raising concerns about its indication.

**METHODS:**

Patients aged <70 years with radiologically solid-dominant clinical stage IA NSCLC ≤2 cm who underwent lobectomy or segmentectomy at three institutions between 2010 and 2017 were enrolled. Propensity scores were estimated to adjust for confounding variables (age, sex, smoking history, tumour location, size, ground-glass opacity, maximum standardized uptake value and histological type). To elucidate the prognostic impact of surgical indications in the late postoperative phase, restricted mean survival time (RMST) from 0 to 5 and 8 years was also determined.

**RESULTS:**

Overall, 388 patients with a median age of 63 years were enrolled. Overall survival (OS) (hazard ratio [HR], 0.447; 95% confidence interval [CI], 0.152–1.316) and recurrence-free survival (RFS) (HR, 0.638; 95% CI, 0.335–1.216) did not differ significantly between the segmentectomy (*n* = 114) and lobectomy groups (*n* = 274). In the propensity score matching of 100 pairs, OS (HR, 0.577; 95% CI, 0.162–2.056) and RFS (HR, 0.945; 95% CI, 0.408–2.191) were comparable between the segmentectomy and lobectomy groups. Regarding OS in the segmentectomy and lobectomy groups, the 5- and 8-year RMST were 4.95 years versus 4.92 years (difference: 0.02 years; 95% CI, −0.09–0.13; *P* = 0.699) and 7.82 years versus 7.69 years (difference: 0.12 years; 95% CI, −0.17–0.42; *P* = 0.420), respectively.

**CONCLUSIONS:**

Segmentectomy is a viable option for younger patients with early-stage NSCLC, suggesting that indications for segmentectomy need not vary by age.

## GRAPHICAL ABSTRACT

**Figure:**
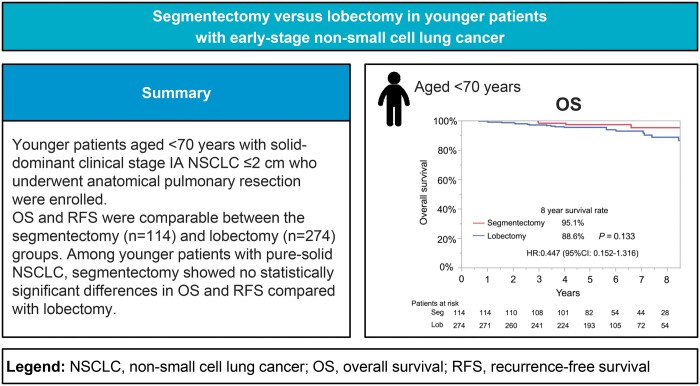

## INTRODUCTION

Lobectomy has been the recommended surgical procedure for early-stage non-small cell lung cancer (NSCLC) since a randomized trial was published by the Lung Cancer Study Group [1]. However, the efficacy of sublobar resection has been demonstrated in recent clinical trials, including the Japanese Clinical Oncology Group (JCOG) 0802/West Japan Oncology Group (WJOG) 4607 l and Cancer and Leukemia Group B 140503, comparing the surgical results between lobectomy and sublobar resection for early-stage peripheral invasive NSCLC sized ≤2 cm [2, 3]. Sublobar resection is considered less invasive than lobectomy, with cancer curability for peripherally located early-stage NSCLC ≤2 cm. Accordingly, many patients with early-stage NSCLC are expected to be treated with sublobar resection. Furthermore, ongoing clinical trials have investigated the possibility of further expanding the application of segmentectomy to larger and more invasive NSCLCs [4].

Although segmentectomy had survival benefits regardless of age in a subgroup analysis of the JCOG0802/WJOG4607L trial, a relatively higher survival benefit of segmentectomy on overall survival (OS) was observed in patients aged ≥70 years than in those aged <70 years [2]. In addition, in a supplemental analysis involving patients with pure solid NSCLC, recurrence-free survival (RFS) following segmentectomy tended to be inferior to that after lobectomy in patients aged <70 years, despite the demonstrated efficacy of segmentectomy on OS [5]. Considering the risk of recurrence following segmentectomy, there is controversy regarding the indications for segmentectomy in younger patients. Generally, younger patients have better general function than older patients [6–8]. Therefore, younger patients aged <70 years may obtain relatively lower survival benefits in the early postoperative period owing to the preservation of the lung parenchyma by segmentectomy compared with older patients. The prognostic impact of lung parenchyma preservation may be greater in the late postoperative period (that is, as younger patients get older). However, this remains unclear. Although appropriate surgical procedures for older patients have been widely discussed [9], the survival benefits of segmentectomy in younger populations have not been well described in previous studies.

Therefore, this study aimed to compare the postoperative long-term outcomes of segmentectomy and lobectomy in younger patients with early-stage NSCLC using propensity score-matched analysis and elucidate the prognostic impact of surgical indications in the late postoperative phase.

## PATIENTS AND METHODS

### Ethics statement

The institutional review boards of the participating institutions approved the retrospective review of a prospective database and waived the requirement for informed consent (Hiroshima University Hospital: 30 November, 2022, approval number: E2018-1216–02; Kanagawa Cancer Center: 3 October 2022, approval number: 24-KEN-54; Tokyo Medical University Hospital: 10 February 2023, approval number: SH2969).

### Study design and patient population

**Figure 1:**
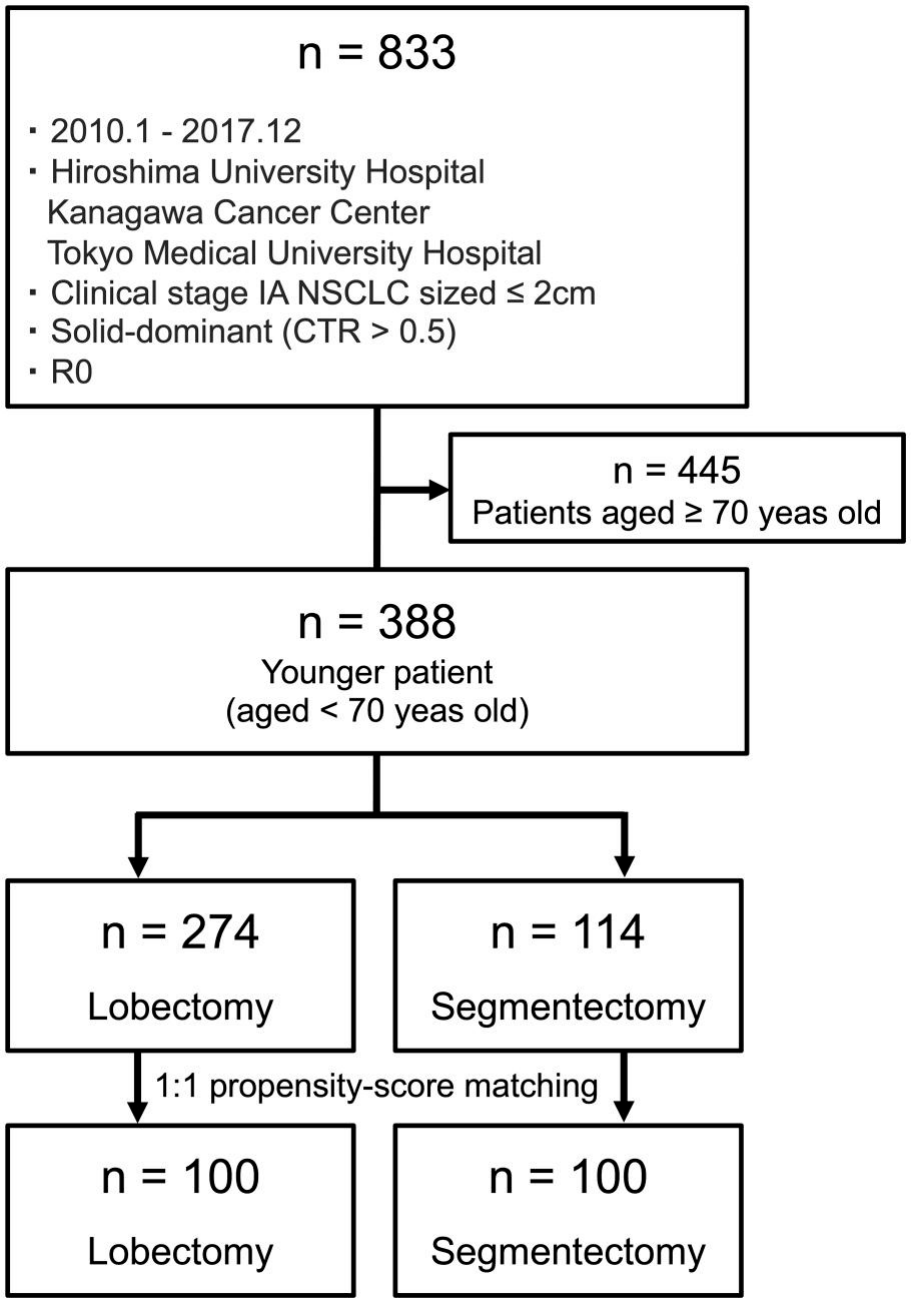
Flowchart of patient selection. The preoperative staging was based on the TNM classification of malignant tumours, eighth edition

As in several previous reports, the cut-off age between younger and older patients was 70 years [2, 3, 6, 7, 10, 11]. Data were obtained from consecutive younger patients, defined as those aged <70 years, diagnosed with radiologically solid-dominant clinical stage IA NSCLC sized ≤2 cm on preoperative high-resolution computed tomography (HRCT). These patients underwent segmentectomy or lobectomy without induction therapy at Hiroshima University Hospital, Kanagawa Cancer Center and Tokyo Medical University between January 2010 and December 2017. Data were obtained from medical records and retrospectively analyzed (Fig. 1). An individual-level database was maintained. The preoperative staging was based on the HRCT findings of 18-fluoro-2-deoxyglucose positron emission tomography/computed tomography (FDG-PET/CT). Preoperative endobronchial ultrasonography and mediastinoscopy were not performed routinely. After excluding patients with tumours in the right middle lobe, survival outcomes after segmentectomy and lobectomy were compared (Fig. 1).

The tumour-node-metastasis classification of malignant tumours (eighth edition) was used for tumour staging [12]. Pathological diagnosis was based on the World Health Organization Classification of Tumors of the Lung, Pleura, Thymus, and Heart [13]. Lymph node metastasis was considered negative when swollen mediastinal or hilar lymph nodes >1 cm in short-axis diameter were not detected on HRCT and when there was no accumulation of FDG in these nodes on FDG-PET/CT images.

### HRCT and FDG-PET imaging

Chest images were acquired using a 16-row multidetector CT. High-resolution images were acquired using the following parameters: 120 kVp, 200 mA, section thickness of 2 mm, pixel resolution of 512 × 512, scan duration of 0.5–1.0 s, a high spatial reconstruction algorithm with a 20-cm field of view, and mediastinal (window level, 40 Hounsfield units [HU]; window width, 400–139 HU) and lung (window level, −600 HU; window width, 1600 HU) window settings. Radiologists from each participating institution reviewed all CT images to determine tumour sizes.

For FDG-PET/CT, an anthropomorphic body phantom that conformed to the National Electrical Manufacturers Association standards was used to minimize variability in the maximum standardized uptake value (SUV_max_), which could have resulted from differences in preparation procedures, scan acquisition, image reconstruction and data analysis among the three study centres [14].

### Surgical procedure

Radical segmentectomy and lobectomy, with lymph node dissection and sampling, were performed. The surgical approach and procedures were decided at a surgical conference based on tumour location and patient status.

### Follow-up evaluation

All patients were followed up from the day of surgery. Postoperatively, physical examinations and chest radiography were performed every 3 months, and CT scans were performed every 6 months for the first 2 years. Thereafter, physical examinations and chest radiography were performed every 6 months, and CT was performed annually, even after 5 years postoperatively. Recurrence was determined based on radiographic features or histological evidence. Loco-regional recurrence was defined as tumour recurrence in the preserved lobe, intrathoracic cavity, ipsilateral hilar lymph nodes or mediastinal lymph nodes. Other types of recurrences were defined as distant recurrences. The data cut-off date was 15 September 2022.

### Statistical analysis

Data on patient characteristics are reported as proportions, medians and interquartile ranges (IQRs). Chi-squared, Fisher’s exact and Wilcoxon rank-sum tests were used to compare the data for the segmentectomy and lobectomy groups. Fisher’s exact test was utilized for categorical variables in small sample sizes. McNemar’s test for categorical variables and paired *t*-test for continuous variables were used to analyze propensity-matched patient pairs.

OS was defined as the time from surgery to death from any cause or censorship at the last follow-up. RFS was defined as the time from surgery to recurrence, death from any cause or censorship at the last follow-up. Survival data were estimated using the Kaplan–Meier method, compared using the log-rank test and expressed as hazard ratio (HR). Multivariable Cox proportional hazards regression analysis was used to evaluate the association between surgical procedures and OS/RFS in a unmatched cohort based on preoperative factors, including age (continuous), sex (female/male), smoking history (never/ever), tumour location (upper/lower), solid tumour size (continuous), CT findings (part-solid/pure solid), maximum standardized uptake value (SUV_max_) (continuous) and histological type (adenocarcinoma/others).

The propensity score was estimated using a logistic regression model based on the same factors as those included in the multivariable analysis. Greedy matching with a caliper width of 0.20 standard deviation of the logit transformation for the estimated propensity score was applied. Propensity score matching was performed in a 1:1 ratio using the estimated propensity scores. Standardized differences were calculated to investigate the balance in patient characteristics in the two groups. Survival data in the propensity-score matched cohort were estimated using stratified log-rank tests. Restricted mean survival time (RMST) from 0 to 5 and 8 years was also estimated; the statistical analysis of their difference between the groups was based on an asymptotic normal distribution.

The risk of recurrence, defined as the cumulative incidence of recurrence (CIR), was estimated using a cumulative incidence function that accounted for death without recurrence as a competing event.

Statistical significance was set at *P*<0.05. All statistical analyses were performed using JMP (SAS Institute, Cary, NC, USA) version 16 and EZR version 1.51 (Saitama Medical Center, Jichi Medical University, Saitama, Japan), which is a graphical user interface for R (The R Foundation for Statistical Computing, Vienna, Austria).

## RESULTS

### Patient characteristics

Table 1 lists the characteristics of 388 patients aged <70 years with radiologically solid-dominant clinical stage IA NSCLC sized ≤2 cm who underwent segmentectomy (*n* = 114) or lobectomy (*n* = 274) (Fig. 1). No significant differences were observed in the frequency of segmentectomy and lobectomy across decades (Supplementary Material, Table S1). Significant differences in tumour location (*P* = 0.021), solid tumour size (*P*<0.001), SUV_max_ (*P*<0.001), lymph node metastasis (*P* = 0.035) and adjuvant therapy (*P* = 0.015) were observed between the segmentectomy and lobectomy groups.

**Table 1:** Characteristics of patients regarding segmentectomy and lobectomy in the unmatched and matched cohorts

Variables^a^	Unmatched cohort			Propensity score-matched cohort			
	Segmentectomy (*n* = 114)	Lobectomy (*n* = 274)	*P*	Segmentectomy (*n* = 100)	Lobectomy (*n* = 100)	*P*	SD^b^
Age, years	64 [58–67]	62 [57–66]	0.116	65 [58–67]	64 [57–67]	0.740	0.028
Sex, male	66 (57.9)	151 (55.1)	0.654	55 (55.0)	51 (51.0)	0.671	0.076
Smoking history, ever	68 (59.7)	155 (56.6)	0.652	58 (58.0)	54 (54.0)	0.669	0.072
Tumour location^c^			0.021			0.983	
RUL	80 (48.8)	59 (28.1)		24 (24.0)	23 (23.0)		0.043
RLL	36 (22.0)	50 (23.8)		22 (22.0)	20 (20.0)		0.097
LUL	32 (19.5)	60 (28.6)		34 (34.0)	36 (36.0)		0.058
LLL	16 (9.8)	41 (19.5)		20 (20.0)	21 (21.0)		0.050
Solid tumour size (cm)	1.2 [0.8–1.5]	1.4 [1.1–1.7]	<0.001	1.2 [0.9-–0.5]	1.2 [0.9–1.5]	0.664	0.062
CT findings, pure solid	62 (54.4)	154 (56.2)	0.823	57 (57.0)	52 (52.0)	0.395	0.092
SUV_max_	1.3 [0.7–2.5]	2.2 [1.2–4.6]	<0.001	1.3 [0.8–2.7]	1.7 [0.9–3.1]	0.232	−0.023
Histological type, adenocarcinoma	101 (88.6)	239 (87.2)	0.159	90 (90.0)	88 (88.0)	0.886	0.023
Invasive tumour size, cm	1.1 [0.7–1.5]	1.3 [0.8–1.6]	0.079	1.1 [0.8–1.5]	1.3 [0.7–1.5]	0.541	
Pleural invasion	10 (8.8)	45 (16.4)	0.055	10 (10.0)	8 (8.0)	0.806	
Lymph vessel invasion	19 (16.7)	55 (20.1)	0.481	19 (19.0)	12 (12.0)	0.241	
Vascular invasion	19 (16.7)	70 (25.6)	0.064	16 (16.0)	15 (15.0)	1.0	
Lymph node metastasis			0.035				
N1/N2	1/3 (0.9/2.6)	15/17 (5.5/6.2)		1/3 (1.0/3.0)	2/3 (2.0/3.0)	0.844	
Adjuvant therapy	10 (8.8)	51 (18.6)	0.015	9 (9.0)	10 (10.0)	1.0	

a Categoric data are shown as number (%) and continuous data are shown as median [IQR].

b Standardized differences were provided for variables used for calculating the propensity score.

c Among the unmatched cohort, with 362 available data on tumour location (central or peripheral), 31 (29.3%) tumours in the segmentectomy group and 53 (20.7%) tumours in the lobectomy group were located in the inner two-thirds of the pulmonary parenchyma.

LLL: left lower lobe; LUL: left upper lobe; RLL: right lower lobe; RUL: right upper lobe; SD: standardized difference.

### Comparison of survival outcomes in the unmatched cohort

**Figure 2:**
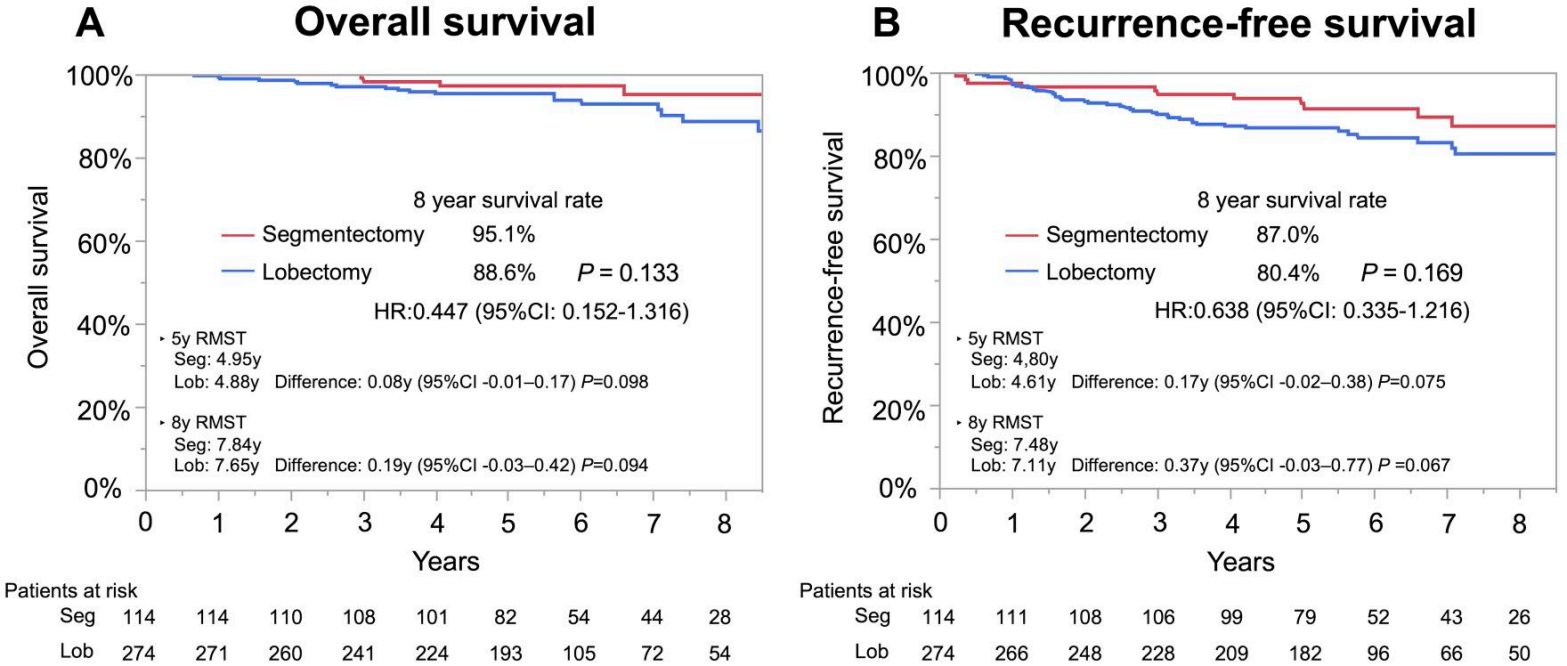
OS and RFS curves in an unmatched younger population who underwent lobectomy or segmentectomy. (**A**) Eight-year OS was 88.6% with lobectomy and 95.1% with segmentectomy (*P* = 0.133). (**B**) Eight-year RFS is 80.4% with lobectomy and 87.0% with segmentectomy (*P* = 0.169)

The median follow-up period was 6.5 (IQR, 5.3–8.2) years. No significant difference in OS (HR, 0.447; 95% confidence interval [CI], 0.152–1.316; Fig. 2A) or RFS (HR, 0.638; 95% CI, 0.335–1.216; Fig. 2B) was observed in the segmentectomy and lobectomy groups. Regarding OS, RMST at 5 and 8 years was 4.95 and 4.88 years in the segmentectomy and lobectomy groups, respectively (difference: 0.08 years; 95% CI, −0.01–0.17; *P* = 0.098). At 8 years, RMST was 7.84 and 7.65 years in the segmentectomy group and lobectomy groups, respectively (difference: 0.19 years; 95% CI, −0.03–0.42; *P* = 0.094). The results of the univariable and multivariable Cox hazard regression analyses for OS and RFS are shown in Table 2. In the multivariable analysis, segmentectomy, compared with lobectomy, was not an independent prognostic factor for OS (HR, 0.389; 95% CI, 0.126–1.204) or RFS (HR, 0.789; 95% CI, 0.405–1.538). The causes of death 5 years postoperatively are shown in Supplementary Material, Table S2.

**Table 2:** Univariable and multivariable analysis for OS and RFS in unmatched cohorts

OS	Univariable			Multivariable^a^		
Variables	HR	95% CI	*P*	HR	95% CI	*P*
Segmentectomy versus lobectomy	0.447	0.152–1.316	0.144	0.389	0.126–1.204	0.102

RFS	Univariable			Multivariable^a^		
Variables	HR	95% CI	*P*	HR	95% CI	*P*
Segmentectomy versus lobectomy	0.638	0.335–1.216	0.172	0.789	0.405–1.538	0.486

a Multivariable analysis was adjusted for age (continuous), sex (female/male), smoking history (never/ever), tumour location (upper/lower), solid tumour size (continuous), CT findings (part-solid/pure solid), SUV_max_ (continuous) and histological type (adenocarcinoma/others).

### Comparison of survival outcomes in the propensity score matched cohort

**Figure 3:**
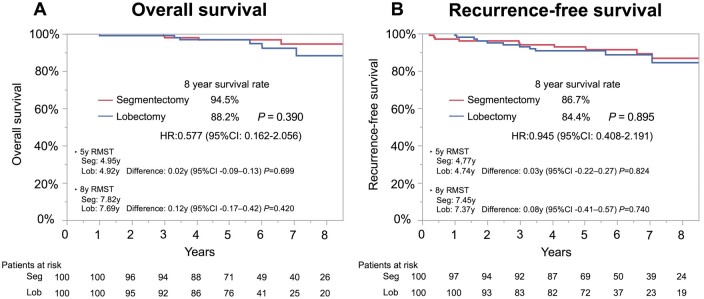
OS and RFS curves in a propensity score-matched younger population who underwent lobectomy or segmentectomy. (**A**) Eight-year OS was 88.2% with lobectomy and 94.5% with segmentectomy (*P* = 0.390). (**B**) Eight-year RFS was 84.4% with lobectomy and 86.7% with segmentectomy (*P* = 0.895)

The characteristics of the propensity score-matched cohorts are listed in Table 1. The clinical variables were balanced between both groups. Among these, OS (HR, 0.577; 95% CI, 0.162–2.056; Fig. 3A) and RFS (HR, 0.945; 95% CI, 0.408–2.191; Fig. 3B) were not significantly different between the segmentectomy and lobectomy groups. Considering OS, the 5-year RMST was 4.95 versus 4.92 years (segmentectomy vs lobectomy; difference: 0.02 years; 95% CI: −0.09–0.13; *P* = 0.699) and the 8-year RMST was 7.82 versus 7.69 years (difference: 0.12 years; 95% CI: −0.17–0.42; *P* = 0.420). Seven (7%) and six (6.0%) patients experienced recurrence in the segmentectomy and lobectomy groups, respectively. CIR was not significantly different between the segmentectomy group (8-year CIR: 8.1%) and the lobectomy group (8-year CIR: 6.2%) (*P* = 0.764). No difference was observed in recurrence patterns between the segmentectomy (loco-regional: 4.0%, distant: 1.0%, unknown: 2.0%) and lobectomy (loco-regional: 5.0%, distant: 0%, unknown: 1.0%) groups (*P* = 0.235; Table 3).

**Table 3:** Relapse pattern and site after surgery in a propensity-score-matched cohort

	Segmentectomy	Lobectomy
	(*n* = 100)	(*n* = 100)
Total relapse	7 (7.0%)	6 (6.0%)
Loco-regional	4 (4.0%)	5 (5.0%)
Surgical stump	0	0
Hilar LN	0	1
Mediastinal LN	1	2
Ipsilateral lung	1	0
Pleural dissemination	2	2
Distant	1 (1.0%)	0 (0%)
Unknown	2 (2.0%)	1 (1.0%)

LN: lymph node.

**Figure 4:**
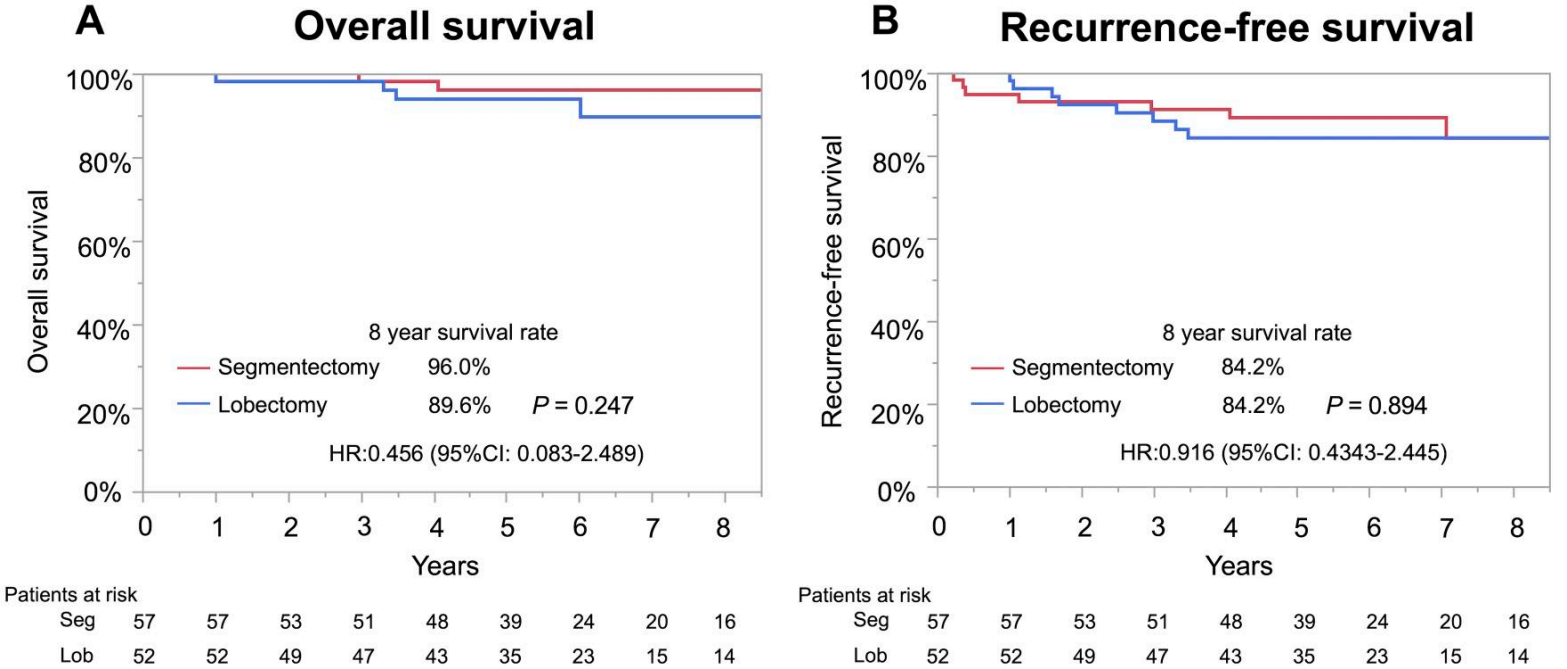
OS and RFS curves for younger propensity score-matched cohort with pure solid NSCLC. (**A**) Eight-year OS was 89.6% with lobectomy and 96.0% with segmentectomy (*P* = 0.352). (**B**) Eight-year RFS was 84.2% with lobectomy and 84.2% with segmentectomy (*P* = 0.861)

Among younger patients with pure-solid NSCLC in propensity score-matched pairs, the segmentectomy group showed no statistically significant differences in OS (HR, 0.456; 95% CI, 0.083–2.489; Fig. 4A) and RFS (HR, 0.916; 95% CI, 0.434–2.445; Fig. 4B) compared with the lobectomy group. Patient characteristics are shown in Supplementary Material, Table S3.

## DISCUSSION

This study compared the long-term (>5 years) postoperative survival in younger patients aged <70 years who underwent lobectomy or segmentectomy for radiologically solid-dominant early-stage NSCLC sized ≤2 cm. Survival outcomes, including OS and RFS, were comparable during the early postoperative periods between younger patients who underwent segmentectomy and those who underwent lobectomy, using propensity score-matched analyses. Furthermore, the difference in RMST regarding OS was slightly larger at 8 years than that at 5 postoperatively, despite showing no statistically significant difference. These results indicated that segmentectomy is a feasible surgical procedure even for younger patients.

Several pivotal clinical trials have demonstrated the significance of sublobar resection in small and early-stage NSCLC [2, 3, 15]. Notably, in the JCOG0802/WJOG4607L trial, segmentectomy showed significantly better OS than lobectomy, regardless of age [2]. Even in a subgroup analysis targeting pure solid lung cancer, considered highly malignant, segmentectomy showed superior OS [2, 5]. However, a further stratified analysis involving patients with pure solid NSCLC and those aged <69 years, OS was slightly better with segmentectomy, but RFS tended to be inferior compared to lobectomy, although patient background information was not provided [5]. Considering the risk of recurrence following segmentectomy, a controversy exists regarding whether the indications for segmentectomy should vary according to age, despite of survival benefits associated with the procedure. Nevertheless, the results of this real-world study revealed that even in pure solid lung cancer, segmentectomy is a feasible and acceptable procedure compared with lobectomy for younger patients, with a tendency towards better OS and comparable RFS.

Owing to its less invasiveness, segmentectomy has contributed to the preservation of postoperative respiratory function [2, 16, 17] and nutritional status [18], as well as reducing the risk of postoperative complications compared with lobectomy [19]. Generally, younger patients have fewer underlying diseases and better performance status and organ function, including pulmonary function, than older patients [6–8]. Therefore, more younger patients can sufficiently tolerate the physical burden of lobectomy. However, younger patients undergoing surgery also experience a decline in systemic function as they age. Previous studies, which included only patients who survived more than 5 years post-surgery, demonstrated that pulmonary function—such as vital capacity and forced expiratory volume in 1 second—was significantly worse after lobectomy compared to segmentectomy during long-term follow-up [20]. Moreover, pulmonary function continued to decrease with age up to 5 years postoperatively. It has been reported that pulmonary function declines with age due to physiological changes, including decreased static elastic recoil of the lung, reduced compliance of the chest wall, diminished respiratory muscle strength, heightened perception of bronchoconstriction and increased heterogeneity of the. ventilation-perfusion ratio [21]. Thus, the survival risk associated with the decline in pulmonary function after lung resection may be more pronounced in the late postoperative period as patients age. Our study also suggested a potential for superior survival following segmentectomy in the late postoperative period, as shown by RMST analysis, despite reaching no statistically significant difference. Although the potential benefit of lung preservation in the early postoperative period may be less for younger patients, it may provide a greater survival benefit, especially in the late postoperative period.

Compared with older patients, lung cancer in younger patients exhibits more malignant and aggressive features [22]. Nevertheless, complete resection is essential, particularly for younger patients [23]. In this study, neither incomplete resection using segmentectomy nor a difference in postoperative recurrence risk, including loco-regional recurrence, was observed between the segmentectomy and lobectomy groups. Furthermore, during long-term follow-up, the risk of death due to primary NSCLC following segmentectomy was not increased in the late postoperative period. In addition, accurate pathological staging has become increasingly important due to recent advances in adjuvant therapy. In the JCOG0802/WJOG4607L trial, no differences were observed in detecting unsuspected nodal involvement [2]. These findings suggest that segmentectomy is a suitable surgical procedure for achieving accurate pathological staging and complete resection.

This study had some limitations. First, due to the retrospective nature of this study, potential confounding factors might not have been completely eliminated. This study resulted in the absence of several indicators for evaluating the efficacy of surgical procedures, such as respiratory function, tumour location, surgical approach, postoperative complication, surgical margin and follow-up rate. Notably, postoperative respiratory function is a critical indicator of systematic function after lung resection. Therefore, further studies are warranted to evaluate the temporal changes in respiratory function and elucidate the impact of lung resection on systemic function in younger patients during the late postoperative phase. Second, this study included segmentectomy with both intensive and passive intent in compromised hosts. Propensity matching did not account for surgical intent, that is, whether segmentectomy was performed with curative or compromised intent. Although cases of segmentectomy with compromised intent may have been present, RFS and OS were similar between the groups. Third, this study may lack sufficient statistical power to detect survival differences owing to the small number of cases of postoperative recurrence or death. Fourth, the concept of ‘older’ is culturally subjective and depends on social, economic and health-related factors. It is necessary to consider chronological age, functional status, frailty and cancer treatment diversity or activity. In this study, we focused on age as a simplified indicator of patient status.

## CONCLUSION

Segmentectomy is a viable surgical option for younger patients with early-stage NSCLC. When complete resection via segmentectomy is feasible, it is unnecessary to adjust the indications for segmentectomy based on age.

## Supplementary Material

ivaf024_Supplementary_Data

## Data Availability

The data supporting this article will be shared upon reasonable request to the corresponding author.

## ABBREVIATIONS

CI: Confidence interval
FDG-PET/CT: 18-Fluoro-2-deoxyglucose positron emission tomography/computed tomography
HR: Hazard ratio
HRCT: High-resolution computed tomography
HU: Hounsfield units
IQR: Interquartile range
JCOG: Japanese Clinical Oncology Group
NSCLC: Non-small cell lung cancer
OS: Overall survival
RFS: Recurrence-free survival
RMST: Restricted mean survival time
SUV_max_: Maximum standardized uptake value
TNM: Tumour-node-metastasis (classification of malignant tumours)
WJOG: West Japan Oncology Group
