# Supplementary material for: Segmentectomy versus lobectomy in younger patients with early-stage non-small cell lung cancer
Source: Interdiscip Cardiovasc Thorac Surg. 2025 Feb 10;40(2):ivaf024. doi: 10.1093/icvts/ivaf024 (PMC11879302; doi:10.1093/icvts/ivaf024)
Supplement: ivaf024_Supplementary_Data [file ivaf024_supplementary_data.zip › Supplementary_Table_S1.docx]

| **Supplementary Table S1. Frequency of segmentectomy and lobectomy by decade** | | |
| --- | --- | --- |
| Year | Segmentectomy (n=114) | Lobectomy (n=274) |
| 2010-2013 | 47 (41.2%) | 124 (45.3%) |
| 2014-2017 | 67 (58.8%) | 150 (54.7%) |
