# Supplementary material for: Segmentectomy versus lobectomy in younger patients with early-stage non-small cell lung cancer
Source: Interdiscip Cardiovasc Thorac Surg. 2025 Feb 10;40(2):ivaf024. doi: 10.1093/icvts/ivaf024 (PMC11879302; doi:10.1093/icvts/ivaf024)
Supplement: ivaf024_Supplementary_Data [file ivaf024_supplementary_data.zip › CORRECT_Supplementary_Table_S2.docx]

| **Supplementary Table S2. Summary of causes of death after 5 years postoperatively in unmatched and matched population.** | | | |
| --- | --- | --- | --- |
| Unmatched cohort | | Segmentectomy (n = 82) | Lobectomy (n = 193) |
| Total | | 1 (1.2%) | 7 (3.6%) |
| Lung cancer death | | 0 | 4 |
| Other death | | 1 | 3 |
|  | Other cancer including second lung cancer | 0 | 1 |
|  | Non-malignant disease | 1 | 2 |
|  | Cardiopulmonary disease | 1 | 1 |
|  | Cerebrovascular disease | 0 | 1 |
| Propensity-score matched cohort | | Segmentectomy (n = 71) | Lobectomy (n = 76) |
| Total | | 1 (1.4%) | 3 (4.0%) |
| Lung cancer death | | 0 | 1 |
| Other death | | 1 | 2 |
|  | Other cancer including second lung cancer | 0 | 1 |
|  | Non-malignant disease | 1 | 1 |
|  | Cardiopulmonary disease | 1 | 0 |
|  | Cerebrovascular disease | 0 | 1 |
