# Supplementary material for: Segmentectomy versus lobectomy in younger patients with early-stage non-small cell lung cancer
Source: Interdiscip Cardiovasc Thorac Surg. 2025 Feb 10;40(2):ivaf024. doi: 10.1093/icvts/ivaf024 (PMC11879302; doi:10.1093/icvts/ivaf024)
Supplement: ivaf024_Supplementary_Data [file ivaf024_supplementary_data.zip › CORRECT_Supplementary_Table_S3.docx]

| **Supplementary Table S3. Patient characteristics of younger propensity score-matched cohort with pure solid NSCLC.** | | | |
| --- | --- | --- | --- |
| Variables^a^ | Segmentectomy | Lobectomy |  |
|  | (n = 57) | (n = 52) | *P* |
| Age, years | 65 [59–68] | 65 [60–67] | .745 |
| Sex, male | 38 (66.7) | 35 (67.3) | 1.0 |
| Smoking history | 38 (66.7) | 37 (71.2) | .681 |
| Tumor location |  |  |  |
| RUL/RLL | 15/10 (26.3/17.5) | 9/8 (17.3/15.4) | .652 |
| LUL/LLL | 22/10 (38.6/17.5) | 24/11 (46.2/21.2) |  |
| Solid tumor size, cm | 1.4 [1.2–1.7] | 1.4 [1.1–1.6] | .397 |
| SUVmax | 2.3 [1.3–3.4] | 2.5 [1.4–4.0] | .377 |
| Histological type, adenocarcinoma | 10 (17.5) | 12 (23.1) | .486 |
| Invasive tumor size, cm | 1.3 [1.0–1.5] | 1.4 [1.0–1.6] | .978 |
| Pleural invasion | 9 (15.8) | 7 (13.5) | .792 |
| Lymph vessel invasion | 17 (29.8) | 10 (19.2) | .267 |
| Vascular invasion | 16 (28.1) | 14 (26.9) | 1.0 |
| Lymph node metastasis |  |  | .793 |
| N1/N2 | 1/3 (1.8/5.3) | 2/3 (3.9/5.8) |  |
| Adjuvant therapy, yes | 9 (9.0) | 10 (10.0) | .801 |
| ^a^ Categoric data are shown as number (%) and continuous data as median (IQR).  Abbreviations: IQR, Interquartile range; LLL, left lower lobe; LUL, left upper lobe; NSCLC, non-small cell lunger cancer; RLL, right lower lobe; RUL, right upper lobe | | | |
